# Are Children With Autism More Likely to Retain Object Names When Learning From Colour Photographs or Black-and-White Cartoons?

**DOI:** 10.1007/s10803-020-04771-2

**Published:** 2020-11-06

**Authors:** Cheriece K. Carter, Calum Hartley

**Affiliations:** Department of Psychology, Lancaster University, Lancashire, LA1 4YF UK

**Keywords:** Autism spectrum disorder, Word learning, Iconicity, Fast mapping, Retention, Pictures

## Abstract

For the first time, this study investigated whether children with autism spectrum disorder (ASD) and typically developing (TD) children matched on language comprehension (*M* age equivalent =  ~ 44 months) are more likely to retain words when learning from colour photographs than black-and-white cartoons. Participants used mutual exclusivity to fast map novel word-picture relationships and retention was assessed following a 5-min delay. Children with ASD achieved significantly greater retention accuracy when learning from photographs rather than cartoons and, surprisingly, responded more accurately than TD children when learning from photographs. Our results demonstrate that children with ASD benefit from greater iconicity when learning words from pictures, providing a data-grounded rationale for using colour photographs when administering picture-based interventions.

Language acquisition is a crucial developmental milestone that underpins children’s cognitive and social development (Carpenter et al. 1998; Tomasello 2003; Vygotsky 1962). Effective communication and engagement with the social world is facilitated by a child’s capacity to learn words, a skill which can be profoundly impaired in autism spectrum disorder (ASD; Tager-Flusberg and Kasari 2013). While the onset of speech in typically developing children (TD) occurs around 12–18 months (Tager-Flusberg et al. 2009; Zubrick et al. 2007), many children with ASD experience delays in acquiring language, often producing their first words around 36–38 months (Anderson et al. 2007; Howlin et al. 2009). Although the majority of individuals with ASD develop functional language skills over the school years (Pickles et al. 2014), approximately 25–30% remain minimally verbal or non-verbal (Bal et al. 2016; Norrelgen et al. 2015; Tager-Flusberg and Kasari 2013). Many of these children are taught to communicate using the Picture Exchange Communication System (PECS; Bondy and Frost 1994), however, research suggests that children with ASD and concomitant language impairments can have difficulty understanding symbolic relationships between words, pictures, and objects (Hartley and Allen 2014a, 2014b, 2015a, 2015b; Preissler 2008). To inform the design and delivery of picture-based interventions, it is essential for research to explore how children acquire vocabulary from pictures. The purpose of the present study is to investigate how ASD impacts children’s ability to learn words from pictures that vary on the dimension of iconicity—the extent to which a symbol resembles its intended referent.

To acquire vocabulary, children must associate the phonology and meaning of a word and form a lasting connection between the two. This process involves identifying a word’s intended meaning (referent selection) and storing the word-referent pairing in memory for later retrieval (retention) and generalisation. Referent selection is complicated by the fact that there are often multiple potential targets for a newly-heard word (Quine 1960) and requires children to direct their attention to a single target (the intended referent). By 2 years of age, TD children can overcome the challenge of referential ambiguity by applying the mutual exclusivity principle (i.e. the assumption that each referent only has a single label; Carey 1978; Markman and Wachtel 1988). Research has investigated children’s use of this lexical heuristic by presenting a single unfamiliar object alongside one or more familiar objects and asking them to identify the referent of a novel word (Horst and Samuelson 2008; Horst et al. 2010). As children already know the name(s) of the familiar object(s), they use mutual exclusivity to deduce that the novel word must refer to the unfamiliar object. However, TD 2-year-olds often forget new words just 5 min after performing at ceiling on mutual exclusivity referent selection tasks (Horst and Samuelson 2008). Thus, it has been proposed that referent selection and retention are underpinned by separate ‘fast mapping’ and ‘slow learning’ processes (McMurray et al. 2012). ‘Fast mapping’ is the ability to rapidly associate a newly-heard word with a novel object by utilising linguistic and non-linguistic cues to figure out *what* the word refers to (Carey 1978). ‘Slow learning’ refers to the activation of associative learning mechanisms that detect and accumulate statistical co-occurrences between words and environmental features over time and contexts (McMurray et al. 2012).

Several early studies attributed word learning difficulties in ASD to children’s reduced responsiveness to social cues that support referent selection, such as gaze and pointing (e.g. Baron-Cohen et al. 1997; Preissler and Carey 2005). However, a growing collection of research shows that many children with ASD can utilise social-communicative cues to inform their referent selection (Bean Ellawadi and McGregor 2016; Hani et al. 2013; Hartley et al. 2020; Luyster and Lord 2009; McGregor et al. 2013). Furthermore, it is well-documented that children spanning the autism spectrum can use lexical heuristics, such as mutual exclusivity, to accurately identify the referents of unfamiliar words (de Marchena et al. 2011; Hartley et al. 2019). These findings raise the possibility that word learning could potentially be scaffolded by presenting visual and linguistic stimuli in a manner that appeals to the strengths of children with ASD. Indeed, supporting referent selection appears to have positive effects on retention, as shown by correlations between these processes in TD children aged 18–30 months (Bion et al. 2013). In Hartley et al. (2019), accuracy on novel referent selection trials predicted children’s receptive vocabulary, suggesting that facilitating referent selection could have long-term benefits for vocabulary acquisition.

While many studies have investigated how ASD influences fast mapping, relatively few have examined retention in this population. Norbury et al. (2010) demonstrated that verbal children with ASD could retain word-object mappings as accurately as TD controls, but recalled fewer semantic details associated with novel words. In two recent papers, Hartley et al. (2019, 2020) found that children with ASD and concomitant language delay retained novel word meanings acquired through fast mapping and cross-situational learning at least as accurately as vocabulary-matched TD controls after a 5-min break. These findings suggest that fundamental mechanisms supporting word learning, and the relationships between them, are not always qualitatively atypical in ASD.

Alongside words, pictures play an important role in supporting language acquisition and communication—they enable learning in the absence of direct experience. Shared picture book reading involves joint attention, pointing, and verbal labelling (Durkin 1995), therefore providing a prime opportunity for children to learn the names of 3-D objects through exposure to their 2-D counterparts (Ninio and Bruner 1978; Tomasello and Todd 1983). Furthermore, augmentative and alternative communication interventions for children with ASD predominantly use pictures to teach functional communication skills and encourage labelling (e.g. PECS; Bondy and Frost 1994). However, in order to learn words from pictures, children must understand that pictures are not the only referents of their associated labels; labels paired with pictures actually refer to independently existing objects that pictures are intended to represent.

From an early age, TD children spontaneously map labels to depicted referents, demonstrating their understanding that pictures are symbolic representations and facilitating the extension of information to real objects (Baldwin 1991; Baldwin et al. 1996; Ganea et al. 2008). Preissler and Carey (2004) examined this ability by teaching 18- and 24-month-olds the name of an unfamiliar object (a whisk) depicted in a black-and-white line drawing. Immediately after mapping, children were asked to identify the referent of the newly-learned word from an array consisting of the previously seen whisk picture and a previously unseen 3-D whisk. Both age groups consistently selected either the real object alone, or both the whisk-picture and the real whisk together. Despite the fact that the picture was the primary stimulus mapped to the word “whisk”, participants did not identify the picture as the sole referent of the label. These findings indicate that young TD children understand referential relationships between words, pictures, and the objects they represent (Ganea et al. 2008; Hartley and Allen 2014a, 2015a).

However, learning in very young TD children is influenced by iconicity. According to Fuller’s (1997) taxonomy, highly-iconic symbols are “transparent” (e.g. colour photographs), moderately-iconic symbols are “translucent” (e.g. black-and-white drawings), and symbols with little or no resemblance to their referents are “opaque” (e.g. written words). Higher levels of perceptual similarity between picture and referent make the symbolic relationship more salient, increasing the likelihood that the viewer will map the correspondence and draw an inference from one to the other (DeLoache 1995). This principle is supported by Ganea et al. (2008) who demonstrated that 15-month-olds could accurately extend labels from highly-iconic photographs to their corresponding objects, but not from less iconic cartoon pictures. Also, the degree that young TD children can successfully imitate a sequence of actions following a picture-book reading interaction is mediated by iconicity (Simcock and DeLoache 2006). When learning words from pictures, greater visual detail may lead to the formation of more robust representations of meaning during word-picture mapping, facilitating recognition of relationships between pictures and their referents when viewed independently (Ganea et al. 2008; Simcock and DeLoache 2006).

Despite widespread use of picture-based interventions to support individuals with ASD, relatively little research has investigated how children with ASD comprehend pictures. To assess understanding of symbolic relationships between words, pictures, and objects, Preissler (2008) taught minimally verbal children with ASD the word “whisk” in association with a black-and-white line drawing of a whisk. Unlike Preissler and Carey’s (2004) TD participants, the majority of children with ASD thought the word “whisk” only applied to the picture and did not extend the label to the symbolised object. This striking difference suggests that minimally verbal children with ASD have difficulty understanding symbolic word-picture-object relationships. These results are especially concerning given that minimally verbal children with ASD are often taught names for 3-D objects via labelling their 2-D counterparts.

However, it is possible that Preissler’s (2008) participants were hindered by the iconicity of the pictorial stimuli. Hartley and Allen (2015a) tested this possibility by examining how minimally verbal children with ASD extend labels from pictures that varied in iconicity. Their findings revealed that participants extended words to objects most accurately when taught using colour photographs. They also showed that children with ASD were more likely to extend names to objects depicted in colour pictures than non-colour pictures. Together, these result suggest that iconicity has an important impact on symbolic understanding of pictures for children with ASD and concomitant language impairments (also see Hartley and Allen 2014a, b, 2015b, c).

A shared feature of the aforementioned studies investigating children’s understanding of word-picture-object relationships is their focus on fast mapping. Children are taught to associate a novel word with an unfamiliar picture and then presented with the opportunity to extend the label to the 3-D referent almost immediately after the word-picture relationship is established. However, this does not necessarily reflect how children learn from pictures in the real world. It is relatively uncommon to see a picture and then immediately encounter its referent; rather, a child might see a picture and hear its label on one occasion, but then be required to retrieve the representational meaning when they experience the symbolised referent on another occasion. For example, a child might first learn the meaning of "aeroplane" while looking at a picture of an aeroplane in a book, but then be required to recall the label shortly after while playing in the garden and seeing a real aeroplane fly overhead. Thus, the present study explores children’s ability to *retain* novel words when learning from pictures and examines how iconicity influences this ability. As there are currently no data-grounded guidelines regarding which kinds of pictures should be used when delivering PECS, this study could strengthen the argument that highly-iconic colour pictures are most appropriate and effective.

The objective of this study is to investigate how ASD influences children’s ability to learn words from pictures that vary in iconicity (e.g. colour photographs vs. black-and-white cartoons). Following a similar procedure as used in Hartley et al. (2019), children with ASD and TD controls mapped novel word-picture relationships in a mutual exclusivity referent selection task. After a 5-min delay, children’s retention of each word-referent pairing was assessed via two trial types. In ‘picture trials’, children were presented with the unfamiliar pictures that were named during the referent selection trials. In ‘object trials’, children were presented with the 3-D referents of pictures that were named during the referent selection trials. Based on previous evidence (e.g. Preissler and Carey 2005), we predicted that both groups of children would correctly fast map new words to unfamiliar pictures in both conditions, but this might not be sufficient to support above-chance retention. In both participant groups, we expected to observe superior retention when learning words from highly-iconic colour photographs in comparison with less-iconic black-and-white-cartoons.

Importantly, this research addresses significant limitations of the existing literature by testing retention of words learned from pictures and probing children’s understanding of what those words actually refer to. Our study is designed to better reflect children’s real-life usage of pictures and bridge the methodological gap between research examining children’s word learning from pictures and objects (Horst and Samuelson 2008; Horst et al. 2010; McMurray et al. 2012). The findings will advance theoretical understanding by highlighting how learning from pictures impacts fast mapping and retention word learning mechanisms in both typical and autistic development, and ascertain the influence of iconicity.

## Method

### Participants

Participants were 20 children with ASD (14 males, 6 females; *M* age = 80.35 months, *SD* = 28.12) recruited from specialist schools, and 20 TD children (8 males, 12 females; *M* age = 41.55 months, *SD* = 7.17) recruited from mainstream nurseries (see Table 1). All children had normal or corrected-to-normal colour vision. Groups were matched on their receptive language skills, as measured by the Receptive Language module of the Mullen Scales of Early Learning (Mullen 1995). The children with ASD had a mean language comprehension age of 43.60 months (*SD* = 14.74) and the TD children had a mean language comprehension age of 44.75 months (*SD* = 8.80), *t*(38) = 0.30, *p* = 0.77. Children’s expressive language abilities were measured using the Expressive Language module of the Mullen Scales of Early Learning (Mullen 1995). The expressive language age equivalents for the children with ASD (*M* = 37.70 months; *SD* = 19.41) and TD children (*M* = 44.95 months; *SD* = 10.52) did not significantly differ, *t*(38) = 1.47, *p* = 0.15. All participants with ASD had previously obtained a clinical diagnosis from a qualified specialist, based on the criteria of standardised instruments (i.e. Autism Diagnostic Observation Schedule Version 2; Lord et al. 2012 and Autism Diagnostic Interview-Revised; Lord et al. 1994, Rutter et al. 2003) and expert judgment. Diagnoses were confirmed via the Childhood Autism Rating Scale Second Edition (CARS-2; Schopler et al. 2010), which was completed by each participant’s class teacher (ASD: *M* score: 35.73, *SD* = 4.38; TD: *M* score: 16.03, *SD* = 2.28). Children with ASD were significantly older (*t*(38) = 5.98, *p* < 0.001, *d* = 1.89), and had significantly higher CARS scores (*t*(38) = 17.86, *p* < 0.001, *d* = 5.64) than the TD children. Children’s non-verbal intellectual abilities were measured using the Leiter-3 (Roid et al. 2013). The mean (age-normed) IQ for the ASD group was 80.45 (*SD* = 13.30) and the mean IQ of the TD group was significantly higher at 102.25 (*SD* = 6.66), *t*(38) = 6.56, *p* < 0.001, *d* = 2.07. However, the groups’ raw scores on the Leiter-3 did not significantly differ (ASD: *M* score: 54.80, *SD* = 16.30; TD: *M* score: 48.25, *SD* = 4.84), *t*(38) = 1.72, *p* = 0.093. All procedures performed in this study were in accordance with the ethical standards of institutional and national research committees. Informed consent was obtained from caregivers prior to their children’s involvement in the research. None of the participants in the present study took part in similar studies recently conducted by the second author (e.g. Hartley et al. 2019, 2020).

**Table 1:** Sample characteristics (standard deviation and range in parentheses)

Population	*N*	Gender	Chron. age (months)	Mullen receptive language age equiv. (months)	Mullen expressive language age equiv. (months)	CARS-2 score	Leiter-3 IQ score	Leiter-3 raw score
TD	20	8 males, 12 females	41.55 (7.17; 30–53)	44.75 (8.80; 28–59)	44.95 (10.52; 26–62)	16.03 (2.28; 15–24)	102.25 (6.66; 87–113)	48.25 (4.84; 40–57)
ASD	20	14 males, 6 females	80.35 (28.12; 48–137)	43.60 (14.74; 22–69)	37.70 (19.41; 3–70)	35.73 (4.38; 30–45.50)	80.45 (13.30; 53–107)	54.80 (16.30; 36–107)

*TD* Typically developing, *ASD* autism spectrum disorder, *CARS-2* childhood autism rating scale version 2

### Materials

**Fig. 1:**
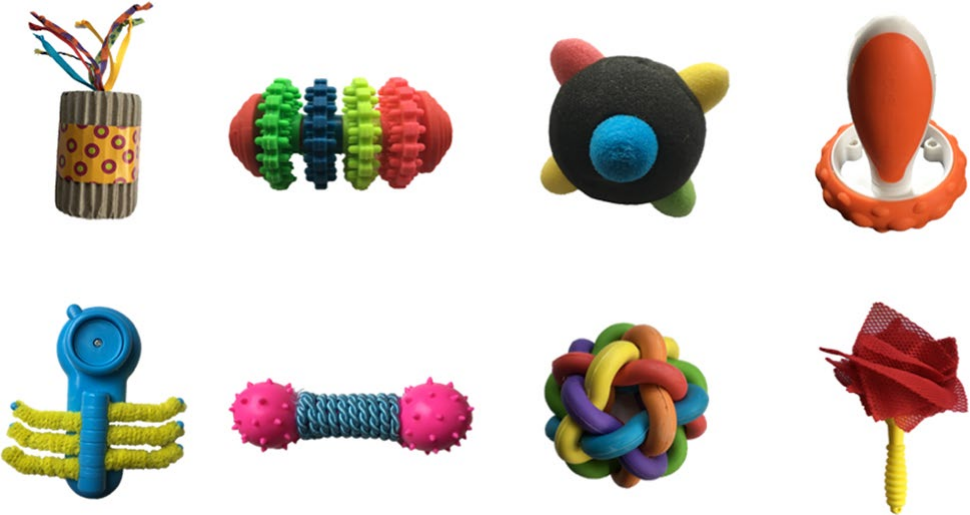
Unfamiliar 3-D objects

Stimuli included eight novel words (parloo, virdex, fiffin, modi, zepper, teebu, nelby, blicket) selected from the NOUN database (Horst and Hout 2016), eight unfamiliar 3-D objects selected on the basis that children would not know their linguistic labels (see Fig. 1), eight corresponding pictures of those unfamiliar objects (four allocated to the Cartoon condition, and four allocated to the Photograph condition), 11 black-and-white cartoon pictures of familiar objects, and 11 colour photographs of familiar objects. All pictures used in this study were laminated and measured 5 cm x 5 cm, which is the recommended sizing for PECS symbols (Frost and Bondy 2002). Pictures of familiar objects were selected on the basis that most children understand their linguistic labels by 16 months (Fensen et al. 1994). The mean age of acquisition for familiar words in the Cartoon (*M* = 12.91) and Photograph (*M* = 12.73) conditions did not significantly differ, *t*(10) = 0.24, *p* = 0.81. A 12-megapixel camera was used to photograph each unfamiliar target object. Three colour photographs of familiar objects were used in the warm-up trials for the Photograph condition (dog, banana, tree). The remaining photographs of familiar objects were divided into pairs and presented alongside photographs of unfamiliar objects in referent selection trials (duck, bed, hand, ball, flower, car, cat, toothbrush). Black-and-white cartoon drawings of familiar and unfamiliar objects were created using Procreate software (version 5.0) on an iPad. Three cartoons of familiar objects were used in the warm-up trials for the Cartoon condition (bottle, key, aeroplane). The remaining cartoons of familiar objects were divided into pairs and presented alongside cartoons of unfamiliar objects in referent selection trials (bird, chair, apple, shoe, balloon, cup, teddy bear, hat).

### Procedure

Our experimental task was very similar to that reported by Hartley et al. (2019). Participants were tested individually in their own educational settings and were accompanied by a familiar adult when required (e.g. key worker, teacher or teaching assistant). Children were verbally praised for attention and good behaviour while completing measures of language comprehension, non-verbal intelligence and word learning. These assessments took the form of fun “games” in separate sessions, on different days.

### Word Learning Task

Children completed two within-subjects conditions—colour photograph and black-and-white cartoon—on different days. Order of administration was counterbalanced within populations (e.g. half of the children with ASD received the Photograph condition first, and half received the Cartoon condition first). Each condition consisted of the following stages delivered in a fixed order: (a) warm-up trials, (b) referent selection trials, (c) test object familiarisation, (d) delay, (e) retention trials. The two conditions only differed in terms of the iconicity of pictures presented during the warm-up and referent selection stages.

#### Warm-Up Trials

Children were informed, “We’re going to play a game where I show you some pictures and ask you to find things…” To begin, all participants completed three warm-up trials; on each trial children were presented with three pictures of familiar objects (e.g. dog, banana, tree) and asked to identify one (e.g. “Which is the tree? Can you see the tree? Show me the tree.”). If children responded correctly, the experimenter issued praise and reinforced the identity of the object (e.g. “Well done, you found the tree!”). If children responded incorrectly, the experimenter provided corrective feedback (e.g. “Actually, this is the tree. Can you point to the tree? Well done, you found the tree!”). The experimenter then retrieved the pictures and re-ordered them for the next trial. Children were asked to identify a different picture in a different location (left, centre, middle) on each trial.

#### Referent Selection Trials

Immediately after the warm-up trials, children completed eight referent selection trials. These followed exactly the same format, except that the experimenter simply said “Thank you…” when children responded, and did not offer praise or corrective feedback. Children were presented with four sets of pictures (each containing one picture of an unfamiliar object, and two pictures of familiar objects). Each set was presented twice; the experimenter requested the familiar object on one occasion (‘familiar trial’; e.g. “Which is the banana? Can you see the banana? Show me the banana.”) and the unfamiliar object on another occasion (‘unfamiliar trial’; “Which is the fiffin? Can you see the fiffin? Show me the fiffin.”). Familiar trials were included to deter children from always choosing the picture of the novel object and encourage them to examine every item in the array. Unfamiliar trials were designed to promote active learning of new word-picture pairings. As participants are likely to have known the labels for the two familiar pictures, they should apply the mutual exclusivity principle and deduce that the novel label refers to the picture of the unfamiliar object (Carey 1978; Markman and Wachtel 1988).

The order of trials was pseudo-randomised with the constraint that the same set of pictures was never presented on consecutive trials and no more than two trials of the same type (familiar or unfamiliar) were experienced sequentially. Positioning of objects (left, middle, right) was pseudo-randomised across trials with the constraint that the requested item did not appear in the same location more than twice consecutively. The four novel words were randomly allocated to the four unfamiliar pictures for each participant. Half of the novel words were always assigned to the Photograph condition (parloo, virdex, fiffin, modi) and the remaining words were always assigned to the Cartoon condition (zepper, teebu, nelby, blicket).

#### Test Object Familiarisation

Immediately after the final referent selection trial, children were familiarised with the as-yet unseen 3-D unfamiliar objects (depicted in the referent selection trials) before their appearance in the subsequent retention trials. The purpose of this stage was to minimise novelty and familiarity preferences, increasing the likelihood that children would select items based on their memory of word-picture mappings. The experimenter presented an unfamiliar 3-D object with a named picture of a different unfamiliar object and then gave a verbal instruction to “look!”. Children were allowed to touch the objects if they wished. After approximately 5 s, the experimenter removed the pair of items from view and presented the next pair until all of the novel objects had been seen. Picture-object pairings were fixed and children received one of two presentation orders. Positioning of items to the left and right was randomised.

#### Delay

Immediately after the final test object familiarisation trial, the experimenter removed all experimental stimuli from the child’s view and initiated a new and unrelated game for 5 min.

#### Retention Trials

**Fig. 2:**
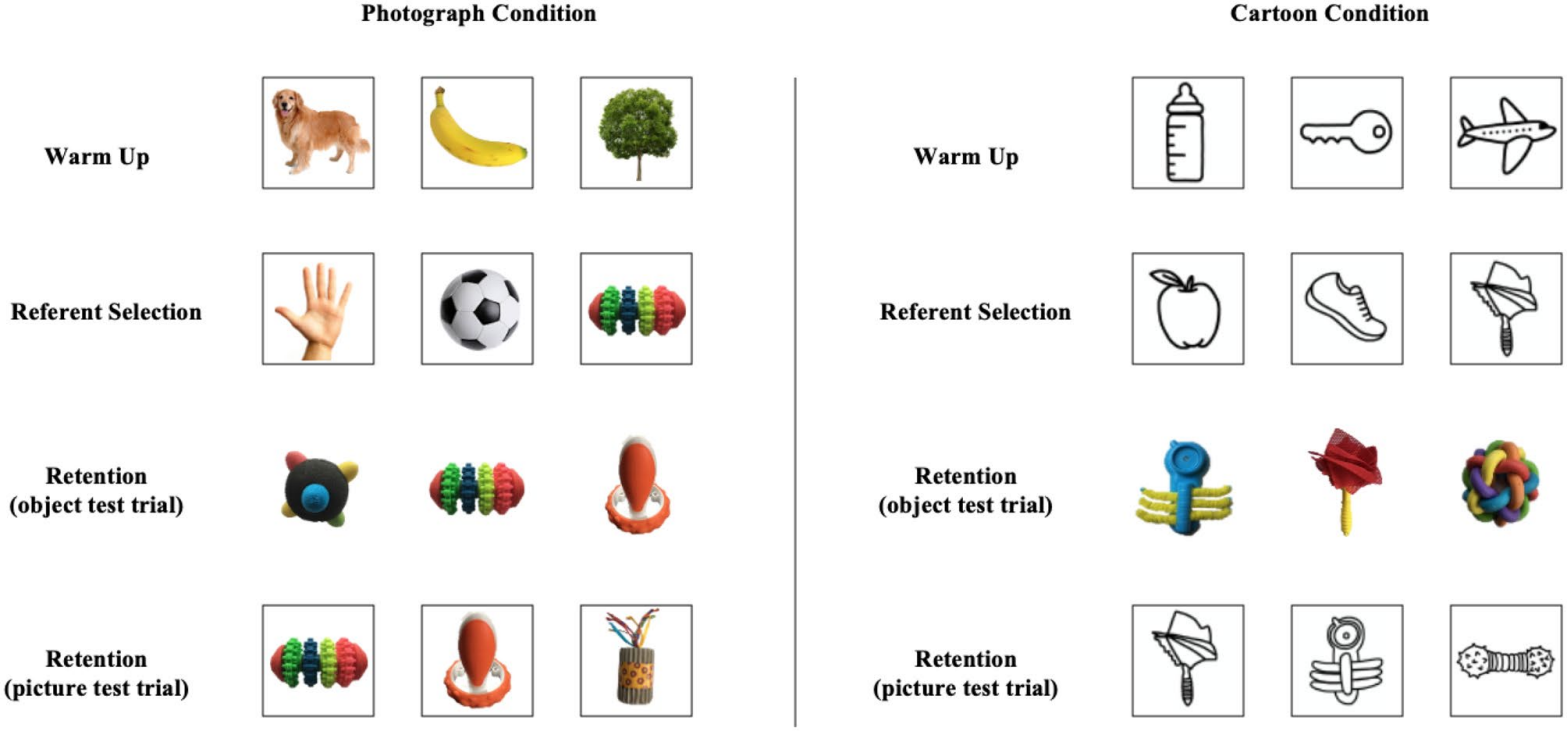
Example stimuli presented at each stage of the experiment in each iconicity condition. Images with black borders represent 2-D pictures, images without black borders represent 3-D objects. The target referent is positioned on the right in the referent selection trial, in the middle on the object retention trial, and on the left in the picture retention trial

To re-engage children’s attention to the task, the experimenter administered one warm-up trial as described above. Eight retention trials immediately followed (see Fig. 2 for an illustration of each trial type). Children’s memory of each word-referent pairing was tested twice (once in each modality: picture and object). For picture retention trials, three pictures of unfamiliar objects that were named during the referent selection trials were presented on the table in front of the child in a row (left, centre, right). The experimenter asked children to identify one of the pictures (e.g. “Which is the parloo? Can you see the parloo? Show me the parloo.”). The purpose of these trials was to assess children’s memory of the exact word-referent pairings that were experienced during the referent selection trials. For object retention trials, three unfamiliar objects that were symbolised by pictures that were named in the referent selection trials were presented on the table in front of the child in a row (left, centre, right). The experimenter asked children to identify one of the objects (e.g. “Which is the virdex? Can you see the virdex? Show me the virdex.”). The purpose of these trials was to assess whether children would extend the novel labels associated with pictures during referent selection to their corresponding 3-D objects.

To provide the necessary level of control when presenting stimuli, object groupings were fixed. Trials were administered in one of four possible orders per condition (evenly allocated across participants in each sample). In each order, no more than two trials of the same type (picture or object) were experienced sequentially and the order of trials was pseudo-randomised with the constraint that the same word was never requested on consecutive trials. Positioning of objects (left, middle, right) was pseudo-randomised across trials with the constraint that the requested item did not appear in the same location more than twice consecutively.

## Results

### Warm Up Trials

In both conditions, all participants achieved 100% accuracy in the warm-up trials.

### Referent Selection Trials

**Fig. 3:**
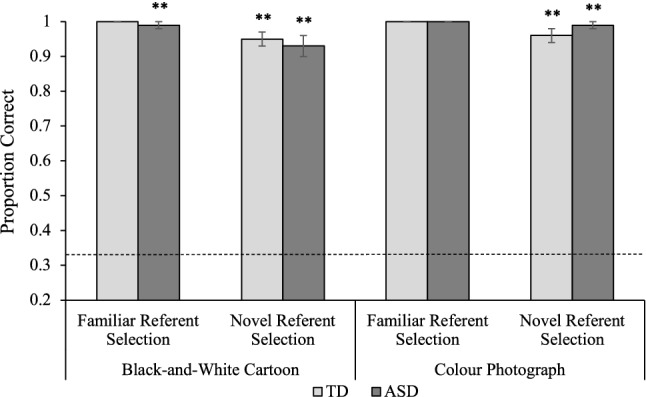
Average referent selection trial accuracy for typically developing (TD) children and children with autism spectrum disorder (ASD). Error bars show ± 1 SE. Dotted line represents chance (0.33). Stars above columns indicate where below-ceiling performance was significantly more accurate than expected by chance (** *p* < 0.001)

Participants were scored out of four on familiar and novel referent selection trials (see Fig. 3). These data were entered into a 2(Population: TD, ASD) × 2(Condition: Cartoon, Photograph) × 2(Trial Type: Familiar, Novel) mixed ANOVA. The analysis revealed a significant main effect of Trial Type, *F*(1,38) = 9.59, MSE = 0.11, *p* = 0.004, η_p_^2^ = 0.20. Accuracy was significantly greater on familiar trials (*M* = 3.99) than novel trials (*M* = 3.83). The analysis also revealed a significant main effect of Condition, *F*(1,38) = 4.18, MSE = 0.07, *p* = 0.048, η_p_^2^ = 0.10. Children achieved significantly greater accuracy on referent selection trials in the Photograph condition (*M* = 3.95) than the Cartoon condition (*M* = 3.86). Despite these significant effects, TD children and children with ASD achieved ceiling level accuracy on familiar and novel trials in both conditions (93–100%), significantly exceeding chance. No other effects or interactions were significant. Pearson’s correlations were conducted to explore relationships between referent selection and measures of individual differences. Although the populations did not differ on accuracy, it is possible that different factors contributed to their successful performance (Happé 1995), so relationships between variables were measured for each population separately. For TD children, there was a significant correlation between accuracy on novel referent selection trials in the Photograph condition and raw score on the Leiter-3, *r*(18) = 0.47, *p* = 0.04 (see Table 2). No significant correlations were observed for children with ASD.

**Table 2:** Correlations between TD trial performance and individual difference measures (^*^ = *p* < 0.05)

	Cartoon ref. sel. novel	Photo ref. sel. novel	Cartoon retention avg	Photo retention avg	Chron. age	Receptive lang	Expressive lang	Non-verb. raw score	Non-verb.IQ
Cartoon ref. sel. novel	–	0.14	0.08	− 0.05	0.06	− 0.07	0.07	0.37	0.10
Photo Ref. sel. novel	*–*	–	0.00	− 0.01	− 0.07	0.25	0.07	0.47^*^	0.41
Cartoon retention avg	–	–	–	− 0.14	0.24	0.40	0.06	− 0.27	− 0.31
Photo retention avg	–	–	–	*	0.40	− 0.05	0.25	0.23	− 0.37

### Retention Trials

**Fig. 4:**
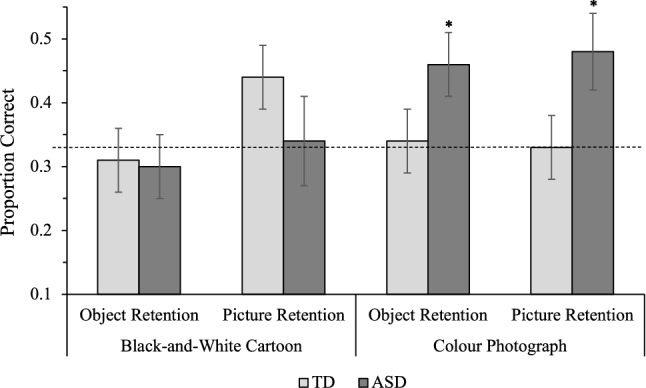
Average retention trial accuracy for typically developing (TD) children and children with autism spectrum disorder (ASD). Error bars show ± 1 SE. Dotted line represents chance (0.33). Stars above columns indicate where below-ceiling performance was significantly more accurate than expected by chance (* *p* < 0.05)

Participants were scored out of four on picture trials and out of four on object trials (see Fig. 4). For picture trials, children responded correctly if they selected the picture that was assigned the requested label during the referent selection trials. For object trials, children responded correctly if they selected the object represented by the picture that was assigned the requested label during the referent selection trials. These data were entered into a 2(Population: TD, ASD) × 2(Condition: Cartoon, Photograph) × 2(Trial Type: Picture, Object) mixed ANOVA. The analysis revealed no significant main effects. The Condition x Population interaction was significant, *F*(1,38) = 4.57, MSE = 1.31, *p* = 0.039, η_p_^2^ = 0.11, and was explored via a series of Bonferroni-adjusted pairwise comparisons. In the Cartoon condition, retention trial accuracy of TD children (*M* = 1.50) and children with ASD (*M* = 1.28) did not significantly differ (*p* = 0.39), however, in the Photograph condition, children with ASD achieved significantly greater accuracy on retention trials (*M* = 1.88) than TD children (*M* = 1.33; *t* = 2.55, *p* = 0.013). Whereas the accuracy of TD children did not significantly differ in the Cartoon (*M* = 1.50) and Photograph (*M* = 1.33; *p* = 0.47) conditions, children with ASD achieved significantly greater retention accuracy in the Photograph condition (*M* = 1.88) than the Cartoon condition (*M* = 1.28; *t* = 2.20, *p* = 0.04). No other interactions were significant. Pearson’s correlations were conducted to explore relationships between retention and measures of individual differences for each population separately. There was a significant correlation between the accuracy of children with ASD on retention trials in the Cartoon condition and their raw scores on the Leiter-3, *r*(18) = 0.68, *p* = 0.001 (see Table 3). Notably, there were no significant relationships between novel referent selection accuracy and retention accuracy in either condition for either population.

**Table 3:** Correlations between ASD trial performance and individual difference measures (^*^ = *p* < 0.05)

	Cartoon ref. sel. novel	Photo ref. sel. novel	Cartoon retention avg	Photo retention avg	Chron. age	Receptive lang	Expressive lang	Non-verb. raw score	Non-verb. IQ
Cartoon ref. Sel. novel	–	0.29	0.13	0.27	0.21	0.35	0.43	0.34	0.21
Photo ref. Sel. novel	–	–	0.23	0.42	0.12	0.33	0.13	0.27	0.20
Cartoon retention avg	–	–	–	− 0.20	0.19	0.34	0.39	0.68^*^	0.36
Photo retention avg	*	*	*	*	− 0.18	0.02	− 0.20	− 0.20	− 0.04

## Discussion

This study investigated how iconicity impacts the ability of children with ASD and TD children to learn words from pictures. Both populations used mutual exclusivity to accurately map novel word-picture relationships in both iconicity conditions, but demonstrated substantially reduced retention accuracy. Children with ASD achieved significantly greater retention accuracy in the Photograph condition than the Cartoon condition and, interestingly, responded more accurately on retention trials in the Photograph condition than TD children. Perhaps surprisingly, TD children’s accuracy on picture and object retention trials did not significantly exceed chance in either iconicity condition. These findings suggest that learning words from pictures may be more cognitively challenging for TD children than learning words from objects. Our results also demonstrate that word learning in ASD is facilitated by greater iconicity, providing a data-grounded rationale for using colour photographs when administering picture-based interventions.

In referent selection trials, TD children and children with ASD responded with extremely high accuracy on familiar and novel referent selection trials in both conditions. Despite a significant difference between trial types, children were almost as accurate at identifying referents of novel words (96%) and familiar words (almost 100%). This finding aligns with previous research demonstrating that children across the autism spectrum reliably use mutual exclusivity to overcome the challenge of referential ambiguity when fast mapping novel words (e.g. Preissler and Carey 2005; de Marchena et al. 2011; Hartley et al. 2019). Similarly, despite a statistically significant effect of iconicity, differences in accuracy between conditions were also minimal (98.8% accuracy in the Photograph condition vs. 96.5% accuracy in the Cartoon condition). These data indicate that both groups of children were capable of identifying familiar referents and distinguishing them from unfamiliar referents when learning from either cartoons or photographs. Thus, iconicity had relatively little impact on children’s ability to accurately fast map labels to pictures.

Despite their highly-accurate responding on referent selection trials, both populations showed substantially reduced retention accuracy after a 5-min delay. Indeed, children’s referent selection accuracy did not significantly correlate with their subsequent retention accuracy, suggesting that these word learning processes are subserved by distinct mechanisms. This is broadly consistent with prior research investigating word learning from objects, where children demonstrate relatively poor retention of word meanings established through fast mapping (Horst and Samuelson 2008; Hartley et al. 2019, 2020). However, retention accuracy for children with ASD was clearly mediated by iconicity. Children with ASD responded with significantly greater accuracy in the Photograph condition than in the Cartoon condition and achieved significantly greater accuracy in the Photograph condition than TD controls. The groups did not differ in accuracy in the Cartoon condition, where neither group significantly exceeded chance level accuracy on either picture or object trials. These results advance prior findings that children with ASD benefit from greater iconicity when *mapping* word-picture-object relations (e.g. Hartley and Allen 2015a) by demonstrating that they also *retain* information with greater accuracy when learning from colour photographs.

Contrary to our predictions, greater iconicity facilitated retention of newly-learned words for children with ASD, but not TD children. One possibility is that our ASD sample demonstrated superior retention in the Photograph condition because they were significantly older than the TD children and may have superior phonological working memory skills (Baddeley et al. 1998). Variations in children’s development of phonological working memory may influence retention of verbal information in the short-term and affect the formation of long-term representations of novel phonological material (Pierce et al. 2017; Baddeley and Hitch 2019). However, this explanation seems unlikely as children with ASD did not achieve significantly greater retention accuracy in the Cartoon condition. An alternative, and perhaps more likely, account of our findings relates to differences in visual processing between the two populations. Children with ASD reliably outperform TD controls during visual tasks with brief presentations (Plaisted et al. 1998; O’Riordan et al. 2001), and this superior performance has been attributed to enhanced perceptual processing (Mottron et al. 2006, 2013). According to this theory, neural networks underpinning perceptual processing are highly specialised in autism and elicit local, rather than global, processing of visual information. Parallels can be drawn between our surprising results and Hartley et al.’s (2019) unexpected finding that children with ASD achieved significantly greater retention accuracy than TD children when tested using differently-coloured stimuli 5 min after fast mapping. Hartley et al. (2019) speculate that superior attention to visual features may elicit more robust encoding of word-object associations during fast mapping when exposures are brief. Therefore, it is possible that enhanced perceptual functioning afforded an advantage for children with ASD when learning from more detailed representations of symbolised referents depicted in colour photographs versus less detailed black-and-white cartoons (where no advantage was observed). However, this account is speculative and warrants validation in future research.

Although greater iconicity may facilitate extension of labels from pictures to specific depicted objects for children with ASD, robust symbolic understanding requires children to generalise labels to multiple category members. Indeed, this is an important objective for picture-based communication interventions. However, recent studies of typical development suggest that increased iconicity may inhibit generalisation. Menendez et al. (2020) taught TD undergraduate students about metamorphosis in ladybirds using life-cycle diagrams that varied on iconicity. Although both perceptually ‘rich’ and ‘bland’ diagrams supported learning, the ‘rich’ diagrams impeded students’ ability to generalise newly-acquired knowledge to other insects. The authors suggest that more iconic learning materials may restrict participants’ ability to generalise because they depict a specific, concrete example of a category, compared to less iconic learning materials that could be interpreted more broadly (Sloutsky et al. 2005). These findings raise the possibility that learning via colour photographs may help children with ASD to map one-to-one picture-object relationships whilst potentially impeding their understanding of pictures as representations of categories. Counter to this, Hartley and Allen (2015a) found that minimally-verbal children with ASD (many of whom were recipients of picture-based interventions) generalised labels to differently-coloured objects with greater accuracy when learning from colour photographs and colour cartoons in comparison to black-and-white cartoons and greyscale photographs. Nevertheless, further research exploring the influence of iconicity on children’s generalisation of word-picture relationships is required.

The lack of an iconicity effect for TD children may be due to their general tendency to preferentially encode shape when processing objects (Landau et al. 1988; Samuelson and Smith 2000; Perry and Samuelson 2011) and pictures (Hartley and Allen 2014a, 2015b). As a general learning principle, shape determines what an object is and therefore the label it should receive (Smith 2000). By approximately 24-months, TD children spontaneously extend unfamiliar labels mapped to novel objects and pictures to additional unlabelled referents based on shape, rather than other perceptual cues such as colour or texture (Landau et al. 1988). Importantly, clearly-recognisable shape cues associated with unfamiliar objects were provided in both the Photograph and Cartoon conditions. For children who primarily focus on shape when learning new object names, it is possible that the category-irrelevant perceptual details afforded by photographs (e.g. colour) do not contribute to encoding stronger word-referent associations when fast mapping (indeed, these additional cues may actually be distracting; see Horst et al. 2019).

Given that the TD group failed to achieve above-chance retention accuracy on any trial type, it may be that the challenge of retaining words learned from pictures is inherently more difficult for these children than words associated with objects. In studies that involve learning names for 3-D objects through fast mapping, it is common for TD three-year-olds to achieve above-chance accuracy (e.g. Horst and Samuelson 2008; Horst et al. 2010). In our study, poor performance on picture retention trials is particularly surprising, as children were tested with the exact stimuli that words were mapped to during referent selection, mirroring conventional retention trials in object learning studies (e.g. Hartley et al. 2019). By 24-months, TD children understand that words paired with pictures relate to independently existing referents and accurately extend labels from pictures to objects (e.g. Ganea et al. 2008; Preissler and Carey 2004; Hartley and Allen 2014a, 2015a). Thus, it is likely that our TD participants realised that labels paired with pictures during referent selection actually referred to independently existing referents. Rather, children’s difficulty retaining words associated with pictures could be explained by the scarcity of dimensional and tactile information afforded by 2-D representations in comparison to 3-D objects. Our data may suggest that generating a robust mental representation of an independently existing object from 2-D sensory input, that can be retrieved after a 5-min delay, could be the challenge for TD children when fast mapping from pictures. Although TD children are capable of developing mental representations of symbolised referents (Hartley and Allen 2015b), retaining these representations could be an ability that develops with age, and could potentially lag behind retention of object representations due to scarcity of information provided at encoding.

Our findings emphasise the importance of studying word learning as a system involving fast mapping and retention mechanisms (McMurray et al. 2012). Most notably, a lack of between-population differences during mapping does not necessarily mean that no differences will be observed for retention. While the majority of previous research exploring children’s learning from pictures has focused on mapping and neglected retention, our findings demonstrate a pressing need to measure *both* of these abilities in order to gain a more comprehensive account of picture-based word learning. We recommend that future studies investigating word learning in ASD should follow this approach. Given the possibility that young TD children might not be as adept at learning vocabulary from pictures compared to objects, we also suggest that future research should bear this in mind when considering methodology and the use of picture vs. object stimuli. From a practical perspective, our findings suggest that word learning in ASD is facilitated by greater iconicity, which supports the use colour photographs when administering picture-based interventions in clinical and educational contexts.

Of course, we must reflect on the limitations of this study. Caution must be exercised when attempting to generalise our results across the autism spectrum. As language development in ASD is extremely heterogeneous, it is important to acknowledge that while mutual exclusivity based referent selection may be a strength for most of the population, the retention of word-referent relationships is likely to be extremely varied, especially when learning conditions are less favourable. Therefore, individuals with more severe developmental delay and language impairments than those displayed by our sample may experience weaker retention. It is also important to acknowledge that the strong performance of children with ASD may partly be attributed to the tightly controlled learning conditions; participants were presented with arrays of only three objects, mapping was not dependent on attention to external factors, distractions were minimised, and participants’ response times were unrestricted. It is also plausible that the performance of our participants with ASD may have been facilitated by prior training on picture-based communication interventions. As we did not record details concerning our participants’ intervention histories, we acknowledge the possibility that the ASD group may have performed particularly well in the Photograph condition because they were familiar with communicating via photographs (although the iconicity of images employed by picture-based interventions varies markedly; Bloomberg et al. 1990; Mirenda and Locke 1989). Moreover, accuracy is only one way to measure word learning. As we did not measure response times, it is possible that our ASD sample may have taken longer than TD participants to respond correctly. Hartley et al. (2020) recently found that children with ASD achieved similar accuracy to vocabulary-matched TD controls, but took significantly more time to generate correct responses. Increased processing time could have significant implications for symbolic understanding of pictures in the real world. In more naturalistic language-learning environments, there would be considerably more noise and external distractions, and quite possibly a greater number of familiar and novel objects visible during a naming event. It is conceivable that comparing our populations under such learning conditions could yield very different results (Yurovsky et al. 2013).

In summary, our study has provided the first account of children’s ability to map *and* retain novel object names when learning from pictures that vary in iconicity. Although children with ASD and TD children performed accurately during fast mapping, regardless of iconicity, both populations demonstrated substantially reduced retention accuracy. However, when learning from photographs, rather than cartoons, children with ASD achieved significantly greater retention accuracy and responded more accurately than TD children. Overall, this research informs understanding of word learning in ASD and identifies possible methods of supporting vocabulary acquisition when learning from pictures.

## References

[CR1] Anderson DK, Lord C, Risi S, DiLavore PS, Shulman C, Thurm A, Welch K (2007). Patterns of growth in verbal abilities among children with autism spectrum disorder. Journal of Consulting and Clinical Psychology.

[CR4] Bal V, Katz T, Bishop S, Krasileva K (2016). Understanding definitions of minimally verbal across instruments: evidence for subgroups within minimally verbal children and adolescents with autism spectrum disorder. Journal of Child Psychology and Psychiatry.

[CR5] Baldwin DA (1991). Infants’ contribution to the achievement of joint reference. Child Development.

[CR6] Baldwin DA, Markman E, Bill B, Desjardins RN, Irwin JM, Tidball G (1996). Infants’ reliance on a social criterion for establishing word-object relations. Child Development.

[CR7] Baron-Cohen S, Baldwin DA, Crowson M (1997). Do children with autism use the speaker’s direction of gaze strategy to crack the code of language?. Child Development.

[CR8] Bean Ellawadi A, McGregor KK (2016). Children with ASD can use gaze to map new words. International Journal of Language & Communication Disorders.

[CR9] Bion RA, Borovsky A, Fernald A (2013). Fast mapping, slow learning: disambiguation of novel word-object mappings in relation to vocabulary learning at 18, 24, and 30 months. Cognition.

[CR78] Baddeley A, Gathercole S, Papagno C (1998). The phonological loop as a language learning device. Psychological Review.

[CR79] Baddeley AD, Hitch GJ (2019). The phonological loop as a buffer store: An update. Cortex.

[CR10] Bloomberg K, Karlan GR, Lloyd LL (1990). The comparative translucency of initial lexical items represented in five graphic symbol systems and sets. Journal of Speech, Language, and Hearing Research.

[CR11] Bondy A, Frost L (1994). The Picture exchange communication system. Focus on Autistic Behavior.

[CR12] Carey S, Halle M, Bresnan J, Miller GA (1978). The child as word learner. Linguistic theory and psychological reality.

[CR14] Carpenter M, Nagell K, Tomasello M (1998). Social cognition, joint attention, and communicative competence from 9 to 15 months of age. Monographs of the Society of Research in Child Development.

[CR15] DeLoache JS (1995). Early understanding and use of symbols: the model. Current Directions in Psychological Science.

[CR16] de Marchena A, Eigsti I-M, Worek A, Ono KE, Snedeker J (2011). Mutual exclusivity in autism spectrum disorders: testing the pragmatic hypothesis. Cognition.

[CR17] Durkin K (1995). Developmental social psychology: from infancy to old age.

[CR18] Fensen L, Dale PS, Reznick JS, Bates E, Thal DJ, Pethick SJ (1994). Variability in early communicative development. Monographs of the Society for Research in Child Development.

[CR19] Fuller D (1997). Initial study into the effects of translucency and complexity on the learning of Blissymbols by children and adults with normal cognitive abilities. Augmentative and Alternative Communication.

[CR20] Frost L, Bondy A (2002). The picture exchange communication system training manual.

[CR22] Ganea P, Bloom-Pickard M, DeLoache J (2008). Transfer between picture books and the real world by very young children. Journal of Cognition and Development.

[CR23] Hani HB, Gonzalez-Barrero AM, Nadig AS (2013). Children's referential understanding of novel words and parent labeling behaviors: similarities across children with and without autism spectrum disorders. Journal of Child Language.

[CR24] Happé FG (1995). The role of age and verbal ability in the theory of mind task performance of subjects with autism. Child Development.

[CR25] Hartley C, Allen ML (2014). Generalisation of word-picture relations in children with autism and typically developing children. Journal of Autism and Developmental Disorders.

[CR26] Hartley C, Allen ML (2014). Intentions vs. resemblance: understanding pictures in typical development and autism. Cognition.

[CR27] Hartley C, Allen ML (2015). Symbolic understanding of pictures in low-functioning children with autism: the effects of iconicity and naming. Journal of Autism and Developmental Disorders.

[CR28] Hartley C, Allen ML (2015). Iconicity influences how effectively minimally verbal children with autism and ability-matched typically developing children use pictures as symbols in a search task. Autism.

[CR29] Hartley C, Allen M (2015). Is children’s naming and drawing of pictures mediated by representational status? Evidence from typical development and autism. Cognitive Development.

[CR30] Hartley C, Bird L, Monaghan P (2019). Investigating the relationship between fast mapping, retention, and generalisation of words in children with autism spectrum disorder and typical development. Cognition.

[CR31] Hartley C, Bird L, Monaghan P (2020). Comparing cross-situational word learning, retention, and generalisation in children with autism and typical development. Cognition.

[CR32] Horst JS, Hout MC (2016). The Novel object and unusual name (NOUN) database: a collection of novel images for use in experimental research. Behavior Research Methods.

[CR33] Horst JS, Samuelson LK (2008). Fast mapping but poor retention by 24-month-old infants. Infancy.

[CR34] Horst JS, Scott EJ, Pollard JA (2010). The role of competition in word learning via referent selection. Developmental Science.

[CR35] Horst JS, Twomey KE, Morse AF, Nurse R, Cangelosi A (2019). When object color is a red herring: extraneous perceptual information hinders word learning via referent selection. IEEE Transactions on Cognitive and Developmental Systems.

[CR36] Howlin P, Magiati I, Charman T (2009). Systematic review of early intensive behavioral interventions for children with autism. American Journal on Intellectual and Developmental Disabilities.

[CR37] Landau B, Smith LB, Jones SS (1988). The importance of shape in early lexical learning. Cognitive Development.

[CR39] Lord C, Rutter M, DiLavore PC, Risi S, Gotham K, Bishop SL (2012). Autism diagnostic observation schedule.

[CR40] Lord C, Rutter M, Le Couteur A (1994). Autism diagnostic interview—revised: a revised version of a diagnostic interview for caregivers of individuals with possible pervasive developmental disorders. Journal of Autism and Developmental Disorders.

[CR41] Luyster R, Lord C (2009). Word learning in children with autism spectrum disorders. Developmental Psychology.

[CR42] McGregor KK, Rost G, Arenas R, Farris-Trimble A, Stiles D (2013). Children with ASD can use gaze in support of word recognition and learning. Journal of Child Psychology and Psychiatry.

[CR43] Mirenda P, Locke PA (1989). A comparison of symbol transparency in nonspeaking persons with intellectual disabilities. Journal of Speech and Hearing Disorders.

[CR44] Mullen EM (1995). Mullen scales of early learning.

[CR45] Markman EM, Wachtel G (1988). Children’s use of mutual exclusivity to constrain the meaning of words. Cognitive Psychology.

[CR46] McMurray B, Horst JS, Samuelson LK (2012). Word learning emerges from the interaction of online referent selection and slow associative learning. Psychological Review.

[CR47] Menendez D, Rosengren KS, Alibali MW (2020). Do details bug you? Effects of perceptual richness in learning about biological change. Applied Cognitive Psychology.

[CR48] Mottron L, Bouvet L, Bonnel A, Samson F, Burack JA, Dawson M, Heaton P (2013). Veridical mapping in the development of exceptional autistic abilities. Neuroscience & Biobehavioral Reviews.

[CR49] Mottron L, Dawson M, Soulieres I, Hubert B, Burack J (2006). Enhanced perceptual functioning in autism: an update, and eight principles of autistic perception. Journal of Autism and Developmental Disorders.

[CR50] Ninio A, Bruner J (1978). The achievement and antecedents of labelling. Journal of Child Language.

[CR51] Norbury CF, Griffiths H, Nation K (2010). Sound before meaning: word learning in autistic disorders. Neuropsychologia.

[CR52] Norrelgen F, Fernell E, Eriksson M, Hedvall Å, Persson C, Sjölin M, Gillberg C, Kjellmer L (2015). Children with autism spectrum disorders who do not develop phrase speech in the preschool years. Autism.

[CR53] O’Riordan MA, Plaisted KC, Driver J, Baron-Cohen S (2001). Superior visual search in autism. Journal of Experimental Psychology: Human Perception and Performance.

[CR55] Perry LK, Samuelson LK (2011). The shape of the vocabulary predicts the shape of the bias. Frontiers in Psychology.

[CR56] Pickles A, Anderson DK, Lord C (2014). Heterogeneity and plasticity in the development of language: a 17-year follow-up of children referred early for possible autism. Journal of Child Psychology and Psychiatry.

[CR80] Pierce LJ, Genesee F, Delcenserie A, Morgan G (2017). Variations in phonological working memory: Linking early language experiences and language learning outcomes. Applied Psycholinguistics.

[CR57] Plaisted K, O'Riordan M, Baron-Cohen S (1998). Enhanced visual search for a conjunctive target in autism: a research note. The Journal of Child Psychology and Psychiatry and Allied Disciplines.

[CR59] Preissler MA (2008). Associative learning of pictures and words by low-functioning children with autism. Autism.

[CR60] Preissler MA, Carey S (2004). Do both pictures and words function as symbols for 18- and 24-month-old children?. Journal of Cognition and Development.

[CR61] Preissler MA, Carey S (2005). What is the role of intentional inference in word learning? Evidence from autism. Cognition.

[CR62] Quine WVO (1960). Word and object: an inquiry into the linguistic mechanisms of objective reference.

[CR64] Roid GH, Miller LJ, Pomplun M, Koch C (2013). Leiter international performance scale.

[CR38] Rutter M, Le Couteur A, Lord C (2003). Autism diagnostic interview-revised.

[CR65] Samuelson L, Smith LB (2000). Grounding development in cognitive processes. Child Development.

[CR66] Schopler E, Van Bourgondien ME, Wellman GJ, Love SR (2010). Childhood autism rating scale.

[CR67] Simcock G, DeLoache J (2006). Get the picture? The effects of iconicity on toddlers' reenactment from picture books. Developmental Psychology.

[CR68] Sloutsky VM, Kaminski JA, Heckler AF (2005). The advantage of simple symbols for learning and transfer. Psychonomic Bulletin & Review.

[CR69] Smith LB, Golinkoff R, Hirsh-Pasek K (2000). How to learn words: An associative crane. Breaking the word learning barrier.

[CR70] Tager-Flusberg H, Kasari C (2013). Minimally verbal school-aged children with autism spectrum disorder: the neglected end of the spectrum. Autism Research.

[CR71] Tager-Flusberg H, Rogers S, Cooper J, Landa R, Lord C, Paul R, Rice M, Stoel-Gammon C, Wetherby A, Yoder P (2009). Defining spoken language benchmarks and selecting measures of expressive language development for young children with autism spectrum disorders. Journal of Speech, Language, and Hearing Research.

[CR72] Tomasello M (2003). Constructing a language.

[CR73] Tomasello M, Todd J (1983). Joint attention and lexical acquisition style. First Language.

[CR75] Vygotsky L (1962). Thought and language.

[CR76] Yurovsky D, Smith LB, Yu C (2013). Statistical word learning at scale: the baby's view is better. Developmental Science.

[CR77] Zubrick SR, Taylor CL, Rice ML, Slegers DW (2007). Late language emergence at 24 months: an epidemiological study of prevalence, predictors, and covariates. Journal of Speech, Language, and Hearing Research.

